# Metabolomic Biomarkers in Urine of Cushing’s Syndrome Patients

**DOI:** 10.3390/ijms18020294

**Published:** 2017-01-29

**Authors:** Alicja Kotłowska, Tomasz Puzyn, Krzysztof Sworczak, Piotr Stepnowski, Piotr Szefer

**Affiliations:** 1 Department of Food Sciences, Faculty of Pharmacy, Medical University of Gdańsk, Al. Gen. J. Hallera 107, 80-416 Gdańsk, Poland; pszef@gumed.edu.pl; 2 Laboratory of Environmental Chemometrics, Faculty of Chemistry, University of Gdańsk, ul. Wita Stwosza 63, 80-308 Gdańsk, Poland; t.puzyn@qsar.eu.org; 3 Department of Endocrinology and Internal Medicine, Medical University of Gdańsk, ul. Dębinki 7, 80-211 Gdańsk, Poland; ksworczak@gumed.edu.pl; 4 Department of Environmental Analytics, Institute for Environmental and Human Health Protection, Faculty of Chemistry, University of Gdańsk, ul. Wita Stwosza 63, 80-308 Gdańsk, Poland; piotr.stepnowski@ug.edu.pl

**Keywords:** Cushing’s syndrome, metabolomics, multivariate analysis, biomarkers, steroid hormones, urinary profiling, gas chromatography, linear discriminant analysis

## Abstract

Cushing’s syndrome (CS) is a disease which results from excessive levels of cortisol in the human body. The disorder is associated with various signs and symptoms which are also common for the general population not suffering from compound hypersecretion. Thus, more sensitive and selective methods are required for the diagnosis of CS. This follow-up study was conducted to determine which steroid metabolites could serve as potential indicators of CS and possible subclinical hypercortisolism in patients diagnosed with so called non-functioning adrenal incidentalomas (AIs). Urine samples from negative controls (*n* = 37), patients with CS characterized by hypercortisolism and excluding iatrogenic CS (*n* = 16), and patients with non-functioning AIs with possible subclinical Cushing’s syndrome (*n* = 25) were analyzed using gas chromatography-mass spectrometry (GC/MS) and gas chromatograph equipped with flame ionization detector (GC/FID). Statistical and multivariate methods were applied to investigate the profile differences between examined individuals. The analyses revealed hormonal differences between patients with CS and the rest of examined individuals. The concentrations of selected metabolites of cortisol, androgens, and pregnenetriol were elevated whereas the levels of tetrahydrocortisone were decreased for CS when opposed to the rest of the study population. Moreover, after analysis of potential confounding factors, it was also possible to distinguish six steroid hormones which discriminated CS patients from other study subjects. The obtained discriminant functions enabled classification of CS patients and AI group characterized by mild hypersecretion of cortisol metabolites. It can be concluded that steroid hormones selected by applying urinary profiling may serve the role of potential biomarkers of CS and can aid in its early diagnosis.

## 1. Introduction

Cushing’s syndrome (CS) is a result of body exposure to prolonged pathologic high levels of cortisol (hypercortisolism) which is produced excessively by adrenal glands. Hypercortisolism may be exogenous (caused by chronic treatment with glucocorticoids resulting in iatrogenic CS) or of endogenous cause. The later results from either increased secretion of corticotropin (ACTH) by tumors located in the pituitary gland (Cushing’s disease, 70%) or other glands (15%). The altered glucocorticoid levels can also be caused by ACTH-independent cortisol secretion from adrenal tumors (15%) [1]. The clinical features of CS can vary and include altered fat distribution, especially in the supraclavicular and temporal fossae, proximal muscle weakness, and wide purple striae. The severity of the symptoms is dependent on the degree and duration of glucocorticoid excess [1]. The diagnosis of Cushing’s syndrome bears many difficulties due to the fact that no single, specific pattern of symptoms can be observed for all patients [2]. The standard approaches include the overnight low-dose dexamethasone (DXM) suppression test, 24-h urinary free cortisol (UFC) assay, and late-night salivary cortisol measurement. All of these tests have potential advantages and disadvantages, can give false-positive and false-negative results, and therefore they should be used complementary to each other [1]. Prolonged exposure to cortisol causes visceral obesity, resistance to insulin, hypertension, osteoporosis, hypothyroidism, altered lipid and glucose metabolism, hypercoagulability, and neuropsychiatric disorders [1,2]. If untreated, the mortality of Cushing’s syndrome associated with cardiovascular diseases is 10%–11% [3,4]. Subclinical hypercortisolism is present in cases of incidentally discovered adrenal tumors (incidentalomas) which according to immunochemical tests are diagnosed as so-called non-functioning masses [5]. Therefore, more reliable, specific, and selective methods which concentrate on the assessment of other biomarkers than solely cortisol are required to carry out early diagnosis of Cushing’s syndrome and subclinical hypercortisolism in patients.

Currently, in biomedical and clinical studies one can observe a visible shift relying on the assay of a complex set of compounds which can serve as biomarkers of a disease instead of the analysis of a single marker chemical [6,7,8]. The assessment, also called metabolomic approach or metabolomics, can be based on the examination of an entire set of compounds (non-targeted approach) or the analysis of selected compounds (targeted approach) present in the analyzed sample [9,10]. The bioanalytical data obtained after the application of metabolomic profiling is usually subjected to multivariate interpretation which enables researchers to identify possible biomarkers of a disease [11]. These biomarkers prove their usefulness for early detection and prognosis of the disease. Moreover, the selected compounds can also be applied for the prediction of the response to targeted therapy. Metabolomic approach has been applied with success in many fields of bioanalytical research, including the selection and assessment of biomarkers in various biological fluids obtained from patients suffering from bladder cancer [12], kidney cancer [13], ovarian cancer [14], prostate cancer [15], and oesophago-gastric cancer [16], or examination of the influence of diet [17] and physical exercise [18] on human body. 

Steroid hormones might be the compounds of choice in the case of novel biomarkers selected for Cushing’s syndrome due to the fact that the disease is connected with elevated levels of cortisol, its altered pathway, and, possibly, altered concentrations of its metabolites [19]. Steroid profiling can be carried out for various samples including blood (serum and plasma), saliva, and urine. Urine steroid metabolomics possess many more advantages when compared to the assays conducted in blood and saliva [20]. First of all, the sample collection is non-invasive and therefore does not influence the levels of steroid hormones synthesized as a response to stressogenic conditions as it is in the case of cortisol. The concentration of the compounds is also higher for urine when compared to saliva (in saliva the value is about 1% of the concentration found in serum or plasma) [20]. Moreover, analyses can be carried out for 24-h urine samples which allows for the ability to avoid the effect of circadian rhythms of steroid excretion characteristic for blood samples [21]. Finally, urine contains not only the major steroid hormones but also their metabolites. Thus, the analysis of this biological material also enables the detection of possible shifts connected with steroidogenesis [22,23].

In our previous paper [23] we have shown that steroid metabolomics can be applied to detect slight steroid hormone overproduction in AIs which may be responsible for generating subtle hypercortisolism in patients (possibly subclinical CS). Given the above considerations, the aim of this follow-up study was to undertake the targeted profiling approach and to measure the levels of major urinary steroid hormones in samples from patients diagnosed with Cushing’s syndrome, compare the results with values for healthy individuals (negative controls), and finally to select potential biomarkers of CS with great emphasis on the levels of steroid metabolites. Moreover, in the study we also carried out the analysis of potential marker compounds of subclinical hypercortisolism (silent cortisol hypersecretion sometimes observed for adrenal incidentalomas). In order to achieve this, we analyzed samples from negative controls, samples from patients with Cushing’s syndrome, and patients with non-functioning adrenal incidentalomas to verify if AIs are not really hypersecretory. The applied procedure allowed for the identification of six steroid hormones which differentiated CS patients from healthy individuals (negative controls) and non-functioning AI and could serve as potential biomarkers of the CS and subclinical hypercortisolism.

## 2. Results

**Figure 1:**
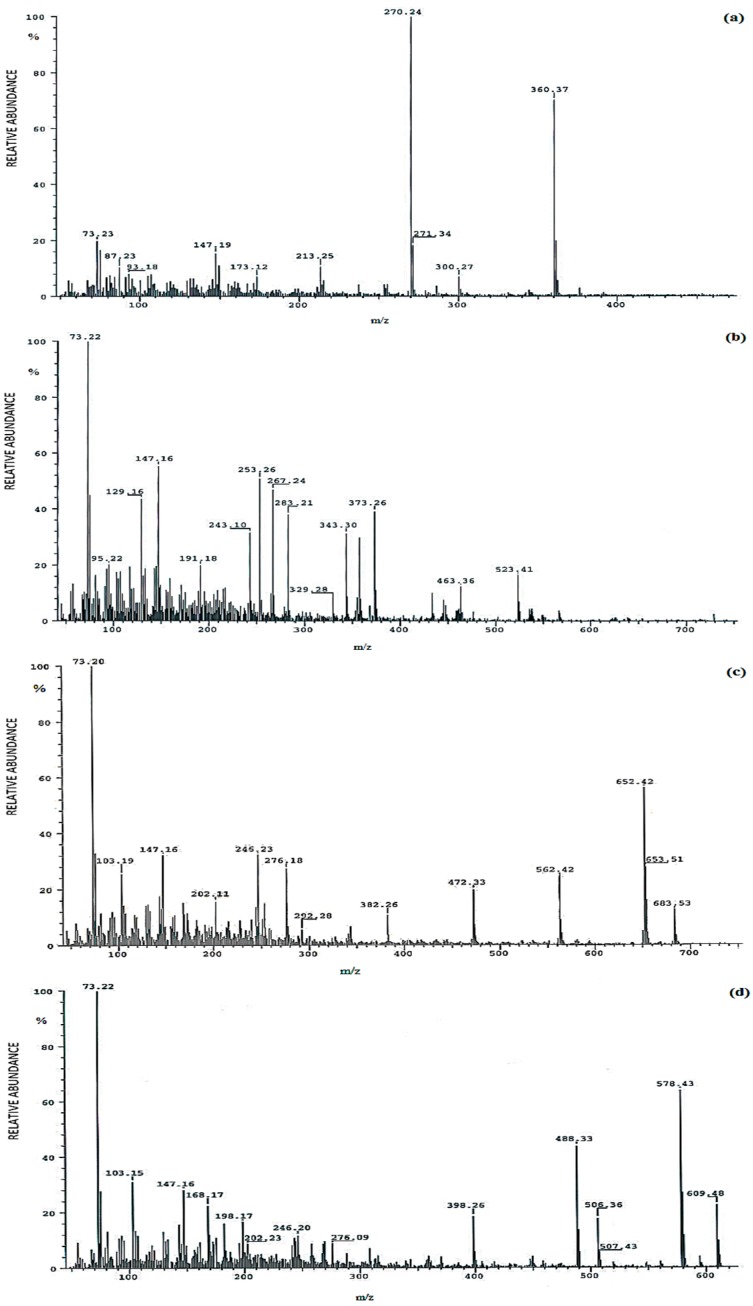
Selected electron ionization (EI) mass spectra of methyloxime-trimethylsilyl ethers (MO-TMS) derivatives of analyzed urinary steroid hormones: (**a**) etiocholanolone; (**b**) α-cortol; (**c**) allo-tetrahydrocortisol; (**d**) tetrahydrocortisone.

Selected electron ionization (EI) mass spectra of methyloxime-trimethylsilyl ethers (MO-TMS) derivatives of analyzed urinary steroid hormones obtained during GC-MS analyses are presented in Figure 1.

The method was linear in the range of 15–200 ng/mL (*R^2^* = 0.997–0.998). Coefficients of variation for intra-day precision (CV, expressed in %) for all analytes ranged from 10.2% to 14.8% for low concentrations and from 5.7% to 9.7% for high concentrations. Inter-day CVs were found in the range of 10.3% to 14.3% for low concentrations and 7.1% to 12.6% for high concentrations (Table 1).

Urinary concentrations of nineteen urinary steroid hormones were determined in examined samples from three study groups. Table 2 illustrates the 24 h excretion of urinary steroid hormones in 78 analyzed samples and the results of ANOVA Kruskal–Wallis followed by Dunn’s test. 

The analysis of the data using ANOVA Kruskall–Wallis test followed by Dunn’s test revealed that urinary concentration of androsterone, etiocholanolone, pregnenetriol, tetrahydrocorticosterone, tetrahydrocortisol, allo-tetrahydrocortisol, and α-cortol was statistically significantly increased for patients diagnosed with Cushing’s syndrome when compared with negative controls and patients with adrenal incidentalomas (*p* < 0.05). Moreover, a decrease in the production of tetrahydrocortisone was also noted for patients with CS (*p* < 0.0001; Table 2). In case of samples obtained from patients with AIs, we have noted a mild, statistically significant increase of the production of etiocholanolone, pregnenetriol, tetrahydrocorticosterone, tetrahydrocortisol, allo-tetrahydrocortisol, and α-cortol and a decrease of tetrahydrocortisone when compared with healthy controls (Table 2). These findings for incidentaloma samples may suggest that the AI patients might suffer from subclinical hypercortisolism. For the assessed levels of other urinary steroids there were no statistical differences noted for the study groups. The results of Mann-Whitney’s *U* test performed for concentrations (mg/24 h urine) of nineteen steroid hormones analyzed in samples from patients with adrenal incidentaloma vs. control and Cushing’s syndrome patients vs. control are presented in Table 3.

The results obtained by applying Mann-Whitney’s test indicated increased levels of tetrahydrocorticosterone, tetrahydrocortisol, allo-tetrahydrocortisol, and α-cortol in samples from AI patients when compared to negative control. Moreover, notable differences in the concentrations of the majority of analyzed compounds (all steroid hormones excluding 11-keto-androsterone, 11-hydroxy-etiocholanolone, androstenetriol, tetrahydro-11-deoxycortisol, and allo-tetrahydrocorticosterone) were noted when Cushing’s syndrome patients were compared with control group.

**Figure 2:**
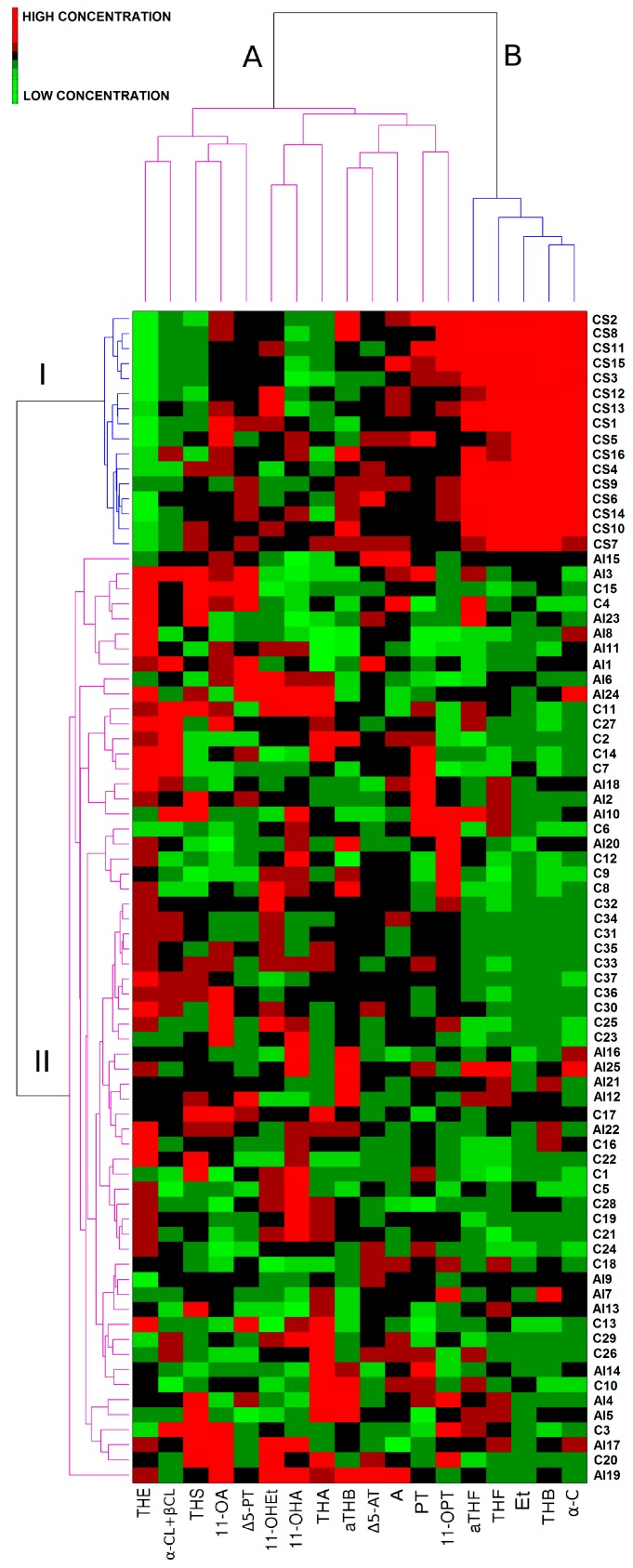
Heat-map generated for urine samples obtained from negative controls (C), patients with non-functioning adrenal incidentalomas (AI), and patients with Cushing’s syndrome (CS). Cluster A groups variables (steroid hormones) which do not generate differences in urinary steroid profiles of all studied individuals, cluster B includes steroid hormones which introduce the highest variability concerning urinary steroid profiles of control (C), and patient groups (AI and CS). Cluster I groups samples obtained from patients with Cushing’s syndrome (CS), cluster II includes samples from negative controls (C), and patients with non-functioning adrenal incidentalomas (AI).

The variations in urinary steroid profiles of patients and negative controls were visualized by pattern recognition methods including a heat-map (Figure 2) and linear discriminant analysis (LDA).

The application of a heat-map analysis revealed grouping of the analyzed samples into two separate clusters (I and II) (Figure 2). The samples were organized on the basis of differences and similarities concerning urinary steroid profiles of examined individuals. For the generated heat-map, color was proportional to log-transformed values of urinary steroid concentration with red representing high and green low concentrations. A clear separation of samples from both negative control and non-functioning adrenal incidentalomas (cluster II) from patients with Cushing’s syndrome could be observed (cluster I). The separation was based on the combination of higher etiocholanolone, tetrahydrocorticosterone, tetrahydrocortisol, allo-tetrahydrocortisol, and α-cortol (variables grouped in separate cluster B) levels as well as lower tetrahydrocortisone concentrations found in urine of patients diagnosed with Cushing’s syndrome when compared to negative control and AI group.

There were twice as many women in the study as men which is associated with the fact that CS is diagnosed more frequently in women than men [5]. To verify the impact of sex on the levels of hormones, confounding effects were studied. Analysis of potential confounders by means of multiple linear regression showed that sex was not a confounding variable in the study (Table 4).

Linear discriminant analysis (LDA) was carried out using experimental data to select potential biomarkers of Cushing’s syndrome. In order to avoid overfitting, the primary step for stepwise LDA was the reduction of the number of variables equal to or less than one third of the total number of studied samples [24]. The analysis of Fisher (*F*), Wilks’–Lambda, and probability (*p*) values enabled the selection of statistically significant steroid hormones which could discriminate between subject classes. Six variables were chosen and two discriminant functions were generated (Equations (1) and (2)).

(1) $$\begin{array}{cc} f_{1}(x)=1.58\;\times & \mathrm{etiocholanolone}-0.46\times \mathrm{tetrahydrocortisone}-0.3\\ & \times \;\mathrm{tetrahydro}-11-\mathrm{dehydrocorticosterone}+1.17\\ & \times \;\mathrm{tetrahydrocorticosterone}+0.6\times \mathrm{tetrahydrocortisol}\\ & +\;0.92\times \alpha -\mathrm{cortol} \end{array}$$

(2) $$\begin{array}{cc} f_{2}(x)=2.15\;\times & \mathrm{etiocholanolone}-0.29\times \mathrm{tetrahydrocortisone}+0.39\\ & \times \;\mathrm{tetrahydro}-11-\mathrm{dehydrocorticosterone}-0.55\\ & \times \;\mathrm{tetrahydrocorticosterone}-1.29\times \mathrm{tetrahydrocortisol}\\ & -\;0.61\times \alpha -\mathrm{cortol} \end{array}$$

**Figure 3:**
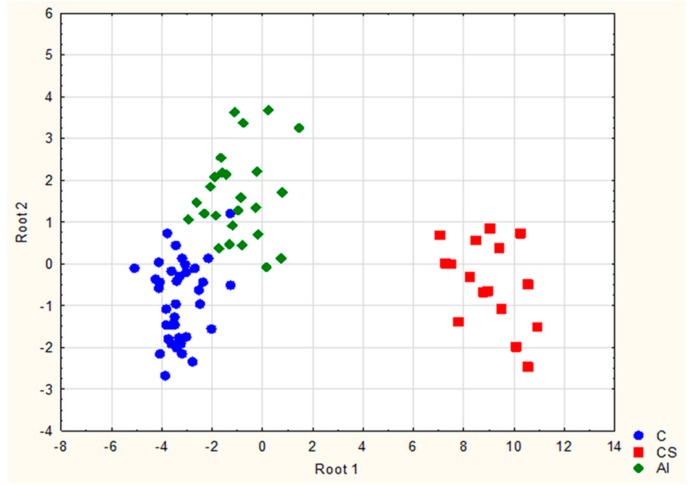
Scatterplot of samples from negative controls (C), patients with non-functioning adrenal incidentalomas (AI), and patients with Cushing’s syndrome (CS) obtained for linear discriminant analysis.

The first function *f*_1_(*x*) enabled the discriminaton of Cushing’s syndrome from healthy individuals (negative control) and patients with adrenal incidentalomas whereas the second *f*_2_(*x*) distinguished AI patients with possible subclinical hypercortisolism from the rest of the study group. Scatterplot of the examined samples corresponding to negative control, adrenal incidentaloma, and Cushing’s syndrome obtained for LDA is presented on Figure 3. 

Clear separation of the three groups could be observed after plotting two discriminatory functions *f*_1_(*x*) and *f*_2_(*x*) (roots) against one another (Figure 3) which suggests that individuals from each group possess different urinary profile and also that selected potential biomarker compounds are highly discriminative. Overall classification rate for the obtained functions was equal to approximately 90% (Table 5). 

For Cushing’s syndrome group the correct classification rate was 100% which proves excellent discriminative power of the obtained classification functions and indicates their high sensitivity and specificity for the detection of the disease. Moreover, high classification rate for adrenal incidentaloma (80%) also proves that the selected six steroids can be viewed as potential biomarkers of subclinical hypercortisolism. The lower values of classification rates for adrenal incidentaloma (80%) and negative control group (approximately 92%) as well as misclassification of these groups may be connected with the fact that in case of mild steroid hormone overproduction, the concentrations of compounds in samples from both groups may be very similar. Moreover, the values for daily excretion of urinary steroids fall within specific ranges and notable inter-individual differences in urinary steroid concentrations can be observed.

The predictive power of the classification functions was verified by conducting out cross-validation applying leave-one-out technique (LOO) [25]. The results of cross-validation show that the method is appropriate for distinguishing and identifying Cushing’s syndrome in examined individuals with accuracy of 100% (Table 6).

## 3. Discussion

Biomarkers commonly assessed in clinical evaluation of Cushing’s syndrome are either salivary, urinary, or serum cortisol [26,27]. Due to the fact that the diagnosis of the disease is often difficult, it is frequently aided by dexamethasone suppression test and ACTH serum levels [27]. However, due to limited sensitivity and specificity of the assays, the results of the steroid hormones evaluation may be inconclusive, and patients suffering from CS may be characterized by steroid levels that represent the upper normal range [28,29,30]. Moreover, subclinical hypercortisolism is also often problematic to diagnose when compared to normal production of cortisol or so-called pseudo-Cushing state, as diagnostic tests can generate false-negative and false-positive results [28]. 

In our study, we chose the metabolomic approach to distinguish and separate patients diagnosed with Cushing’s syndrome from negative controls and patients with adrenal incidentaloma (possible subclinical hypercortisolism) basing on urinary steroid profiling. We have observed that increased urinary levels of androsterone, etiocholanolone, pregnenetriol, tetrahydrocorticosterone, tetrahydrocortisol, allo-tetrahydrocortisol, and α-cortol, as well as decreased concentrations of tetrahydrocortisone, were shown to be specific for Cushing’s syndrome patients, possibly suggesting altered steroid pathways in those individuals. Similar findings were also noted for examined non-functioning adrenal incidentalomas and these results might indicate the presence of subclinical hypercortisolism (excluded during earlier laboratory assessments). As we have previously reported for a study concerning hormonal activity of so-called non-functioning adrenal incidentalomas [22], elevated urinary concentrations of tetrahydrometabolites of cortisol such as tetrahydrocortisol, allo-tetrahydrocortisol, and α-cortol can be related to the existence of hormonal activity of tumors and may be the result of increased synthesis of cortisol and its metabolites [22]. In this study, we have shifted the main aim of the research from detection of hypersecretory activity of adrenal incidentalomas to the evaluation of possible biomarkers of CS and, potentially, of subclinical Cushing’s syndrome. Moreover, in comparison to previous research where only preliminary biomarker selection methods (Principal Component Analysis, Hierarchical Cluster Analysis) were used, we have applied a classification technique (LDA) dedicated to biomarker extraction and classification of unknown samples on the basis of the levels of six selected compounds.

**Figure 4:**
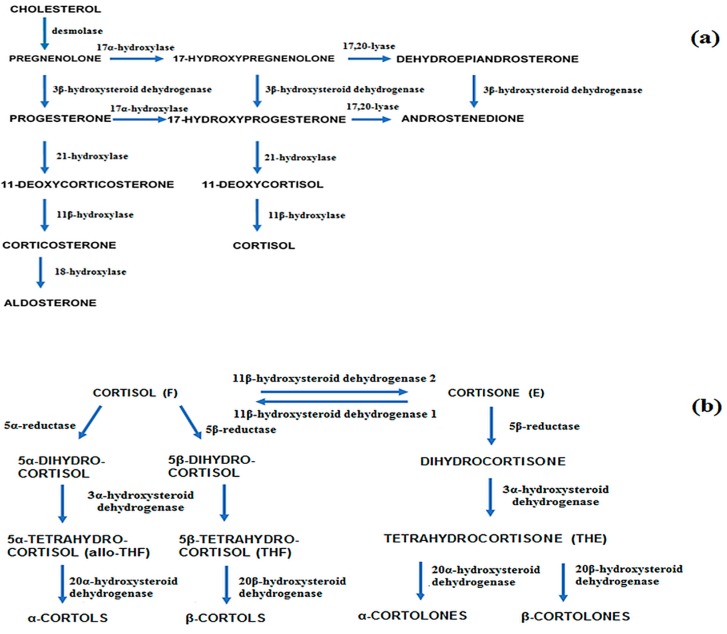
Metabolic pathways of adrenal steroid hormones (**a**) adrenal steroidogenesis; (**b**) principal pathways relating to cortisol metabolism, blue arrows indicate the direction of metabolite flow [36].

There have been previous reports published in literature which highlighted the application of chromatographic analysis of urinary steroids in the search of potential biomarkers of Cushing’s syndrome. However, most studies were focused on the assessment of cortisol and its inactive form (cortisone [31,32,33]) and did not incorporate multivariate data analysis such as LDA or a heat-map. Our approach offers the analysis of multiple potential biomarkers with possibly higher diagnostic power than that of single compounds (e.g., measurement of only cortisol applying immunochemical methods). Our results show that urinary metabolites of cortisol and selected androgens can play the role of discriminating compounds in the diagnosis of Cushing’s syndrome. The applicability of urinary steroid profiling for the diagnosis of CS and the activity of adrenocortical tumors was also tested by Homoki et al. [34]. They reported that on the basis of changes in steroid profiles, CS could be distinguished from healthy, negative controls. Moreover, according to the study, increased levels of free cortisol and its dihydro- and hydroxymetabolites, decreased ratio of tetrahydrocortisone (THE) to tetrahydrocortisol (THF) and increased ratios of THF to allotetrahydrocortisol (a-THF) were characteristic for CS. Kerkhofs et al. also examined the diagnostic value of urinary steroid hormones, however, the study concentrated on the application of the compounds as possible biomarkers of benign and malignant adrenal tumors [35]. Several compounds including metabolites of cortisol, selected androgens, pregnanes, as well as tetrahydro-11-deoxycortisol, proved to be highly specific in discriminating malignant masses. In our study, we have also observed a shift of the synthesis of tetrahydrometabolites of cortisone (THE) to tetrahydrometabolites of cortisol (THF) (Figure 4) [36]. Both compounds play an important part in discriminating CS and possibly subclinical hypercortisolism from negative controls and were therefore selected as potential biomarkers of the diseases after conducting linear discriminant analysis. Similar conclusions concerning the mentioned shift in cortisol production in CS patients were drawn in a research concerning the assessment of the activity of 11β-hydroxysteroid dehydrogenase in Cushing’s syndrome [37]. The enzyme is responsible for the interconversion of cortisol to biologically inactive cortisone. The study linked elevated levels of THF and allo-THF in urine to the presence of CS in patients and indicated impaired activity of the forementioned enzyme. Brossaud et al. also investigated the association of modified cortisol metabolite excretion with the presence of adrenal incidentalomas or Cushing’s disease [38]. The study found that cortisol, tetrahydrocortisol (THF), and tetrahydrocortisone (THE) levels were higher in samples from adrenal incidentaloma patients when compared to control subjects (even though immunoreactive UFC was similar). As a result, cortisol, α- and β-cortolone, and α-cortol were selected as potential biomarkers distinguishing AIs from healthy controls.

The panel of steroid hormones selected as potential biomarkers of CS turned out to be characteristic also for subclinical hypercortisolism associated with hormonal activity of non-functioning adrenal incidentalomas. Recent reports show that for several “clinically silent” adrenal tumors in patients without strong features of Cushing’s syndrome, metabolomic profiling of steroid hormones actually reveals hormonal activity [21,22,39]. The activity may be possibly associated with the potential defect of 11β-hydroxysteroid dehydrogenase activity. This might explain moderately increased levels of tetrahydrometabolites of cortisol and a shift into cortisol overproduction observed for samples from AI patients in this study. It is also worth noting that some researchers associated impaired 11β-hydroxylase deficiency with induction of adrenal tumorgenesis [40].

To conclude, the results of the study demonstrate that selected urinary steroid hormones can serve as potential biomarkers of Cushing’s syndrome and, possibly, subclinical hypercortisolism. The findings also indicate the advantage of using a metabolic approach combined with multivariate analysis in distinguishing subtle metabolic differences between the study groups. However, due to the relatively small number of samples, the clinical impact of our study is limited. Further studies should be therefore carried out on large patient cohorts in order to gain further insights into Cushing’s syndrome biomarkers and to fully validate the selection of compounds using a large, external study sample. 

## 4. Materials and Methods

### 4.1. Ethics Statement

All participants signed informed consents and the study was approved by the Local Ethical Committee of the Medical University of Gdańsk, Poland, identification code NKEBN/274/2009 (approved on 5 October, 2009).

### 4.2. Study Participants

The selection criteria for patients and the negative control group have been previously described [22,23]. The sample cohort for the follow-up study included 37 healthy negative controls (12 men and 25 women), 25 patients with non-functioning incidentalomas (7 men and 18 women), and 16 patients with Cushing’s syndrome excluding iatrogenic CS (5 men and 11 women). All participants were matched in terms of body mass index (BMI) and age (minimum 40 years of age). Information about health status of participants was collected from interviews and medical records. The negative control group included individuals who underwent ultrasonography to eliminate the existence of adrenal tumors, did not use medication which could influence steroidogenesis (induce the expression or activity of cytochrome P450 enzymes), and did not show signs of hypercortisolemia. Individuals suffering from: endocrine or metabolic disorders, increased prolactin levels (hyperprolactinemia), dyslipidemia or hypercholesterolemia, presence of androgen-dependent tumors, hypertension, renal insufficiency, or hepatic deficiency were excluded from the study. Furthermore, the participants were prohibited from: practicing sport, taking medication (including thyroid hormones and anti-thyroid agents) or drugs, taking anabolic and ergogenic compounds, using tobacco products, and consuming alcohol during the study.

The patients with CS and AIs were recruited among patients treated at the Department of Endocrinology and Internal Medicine of Medical University of Gdańsk. The presence of Cushing’s syndrome was confirmed on the basis of clinical evaluation in terms of clinical symptoms (altered fat distribution, wide purple striae, etc.), assessment of 24-h urinary cortisol (normal range 12–330 nmol/24 h), serum cortisol levels after suppression test applying 1 mg dexamethasone (DXM) (immunofluorescent assay, concentrations > 140 nmol/L indicated hypercortisolism), and analysis of plasma ACTH levels (normal range 15–46 pg/mL) [41,42]. Fourteen patients had adrenal Cushing’s syndrome due to adrenal adenomas and two patients suffered from pituitary CS. 

The activity of AIs was assessed by determining UFC in 24-h urine and serum cortisol levels after suppression test with DXM using immunofluorescent assay (concentrations < 50 nmol/L excluded hypercortisolism, intermediate cut-off point of 94 nmol/L indicated subclinical hypercortisolism), dehydroepiandrosterone sulphate (DHEA-S) in serum (normal range equal to 34–430 μg/dL), aldosterone levels in serum and urine, potassium in blood, and methoxycatecholamines in 24-h urine [22,41,42]. The immunochemical assays revealed that AIs were non-hypersecretory. The presence of congenital adrenal hyperplasia (CAH) was excluded for AIs. Fifteen samples from AI patients were obtained from our previous study on adrenal incidentaloma activity detection [23].

Patients with Cushing’s syndrome had elevated levels of UFC ranging from 690–1409 nmol/24 h and median of 905 nmol/24 h (5th percentile 690 nmol/24 h, 95th percentile 1409 nmol/24 h). Negative control and adrenal incidentaloma patients had the UFC values which belonged to the normal range. For control subjects the UFC range was from 71 to 137 nmol/24 h with median 106 nmol/24 h (5th percentile 76 nmol/24 h, 95th percentile 135 nmol/24 h) and for AI patients the range was 86–164 nmol/24 h and median was equal to 112 nmol/24 h (5th percentile 86 nmol/24 h, 95th percentile 144 nmol/24 h) [41]. BMI values for control were in the range 25.3–30.1 kg/m^2^ with median 28.5 kg/m^2^ (5th percentile 25.9, 95th percentile 30), for AI patients they were between 27.7–30.6 kg/m^2^ with median 28.9 kg/m^2^ (5th percentile 27.9, 95th percentile 30.3). Cushing’s syndrome patients were characterized by BMI between 29.9–33 kg/m^2^ with median equal to 30.8 kg/m^2^ (5th percentile 29.9, 95th percentile 33). The summary of patients’ metadata is presented in Supplementary Table S1.

### 4.3. Sample Handling and Chromatographic Analysis

Twenty-four-hour urine samples were collected from negative controls, patients with so-called non-functioning AIs, and patients with Cushing’s syndrome. One hundred milliliter aliquots were immediately frozen at −70 °C until the time of processing. Samples were labelled randomly with numbers.

Prior to analysis, the samples were thawed at room temperature and subjected to validated procedure described in our previous reports [23]. Briefly, steroid hormones were extracted using solid phase extraction (SPE) applying C18 cartridges. Next, enzymatic hydrolysis of steroid hormones was carried out and the compounds were extracted by repeating the SPE procedure. Finally, the analytes were derivatized, yielding methyloxime-trimethylsilyl ethers (MO-TMS). Samples labelled with numbers were next analyzed in random order.

GC/MS analyses enabling structure confirmation of the compounds were carried out using a single-quadrupole Finnigan MAT SSQ 710 mass spectrometer (Bremen, Germany) coupled to a Hewlett Packard 5890 gas chromatograph equipped with 30 m × 0.25 mm × 0.25 μm SolGel-1ms capillary column (SGE Analytical Science, Ringwood, Australia) according to the previously described conditions in publication concerning AI activity detection [23].

The quantitative analyses were conducted on Carlo Erba 8000 TOP gas chromatograph equipped with flame ionization detector. Manual injection was used, the injector temperature was 310 °C, split ratio was 1:10, and sample injection volume was 5 μL. SolGel-1ms column was again used for analyte separation. Argon was used as carrier gas with inlet pressure of 95 kPa. The GC oven temperature programming was started at 150 °C and increased to 200 °C at 4 °C/min, next the temperature was increased, reaching the value of 320 °C at 1 °C/min, and held for 30 min. The detector temperature was 320 °C. In each sample we detected and quantified 19 major urinary steroids: androsterone (A), etiocholanolone (Et), 11-keto-androsterone (11-OA), 11β -hydroxy-androsterone (11β-OHA), 11-hydroxy-etiocholanolone (11-OHEt), pregnanetriol (PT), androstenetriol (Δ5-AT), tetrahydro-11-deoxycortisol (THS), 11-keto-pregnanetriol (11-OPT), pregnenetriol (Δ5-PT), tetrahydrocortisone (THE), tetrahydro-11-dehydrocorticosterone (THA), tetrahydrocorticosterone (THB), allo-tetrahydrocorticosterone (aTHB), tetrahydrocortisol (THF), allo-tetrahydrocortisol (aTHF), α-cortolone (α-CL), β-cortolone (β-CL), α-cortol (α-C). Additionally, coefficients of variation for intra-day and inter-day precision were assessed for all examined compounds on two levels of quality control (low and high concentrations in steroid-free urine). The concentration levels of analytes were analyzed five times a day during six consecutive days. The quantification of the compounds was carried out by applying multiple-point calibration curves with analyte concentrations ranging from 15–200 ng/mL).

### 4.4. Statistical Analysis

Data analyses were performed using Statistica 10 (Statsoft, Tulsa, OK, USA) and Matlab (The MathWorks, Natick, MA, USA). In considering the *p*-value used for analyses, *p* < 0.05 was chosen as significant. Log transformation was applied to the analytical data as the distributions of some urinary steroid concentrations were non-Gaussian. Quantitative results are expressed as medians and interquartile ranges, with the values for α-cortolone and β-cortolone given as a sum. The non-parametric ANOVA Kruskal–Wallis for multiple groups comparison [43], followed by Dunn’s test (all pairwise multiple comparison) was used to compare the concentration of metabolites in the samples obtained from three groups. Mann-Whitney’s *U* test was performed to compare steroid levels in samples from patients with adrenal incidentaloma vs. control and Cushing’s syndrome patients vs. control. In order to visualize the differences in the steroid profiles of the study groups, a heat map was generated. The application of multiple linear regression was applied to analyze potential confounding variables (e.g., sex) in the study. Linear discriminant analysis was used to separate the subjects and to identify potential biomarkers responsible for the group differentiation [44]. The analysis was conducted using a forward stepwise variable selection method to construct the predictive models. The obtained classification functions were subjected to cross-validation.

## Figures and Tables

**Table 1:** Intra- and inter-day precisions of analyzed steroid hormones in steroid-free urine.

Standard Applied for Quantification	Intra-Day Precision CV ^1^ (%), *n* = 5		Inter-Day Precision CV ^1^ (%), *n* = 6	
	Low (15 ng/mL)	High (100 ng/mL)	Low (15 ng/mL)	High (100 ng/mL)
Androsterone	11.4	6.2	12.6	10.8
Etiocholanolone	12.3	7.3	12.9	10.2
11-Keto-androsterone	10.2	5.7	12.1	9.3
11-Hydroxy-androsterone	11.5	6.1	10.3	8.4
11-Hydroxy-etiocholanolone	11.4	6.0	10.8	7.9
Androstenetriol	13.2	8.1	12.8	9.9
Pregnanetriol	12.8	8.0	13.5	12.4
Tetrahydro-11-deoxycortisol	10.8	6.9	12.2	9.9
11-Keto-pregnanetriol	13.2	6	12.2	11.4
Pregnenetriol	13.6	7.9	14.2	10.9
Tetrahydrocortisone	13.1	7.5	13.9	12.5
Tetrahydro-11-dehydrocorticosterone	11.5	6.1	12.4	11.2
Tetrahydrocorticosterone	11.3	6.2	12.9	10.6
allo-Tetrahydrocorticosterone	13.4	7.9	11.7	10.3
Tetrahydrocortisol	14.2	8.3	14.3	12.6
allo-Tetrahydrocortisol	13.6	8.7	12.8	10.7
α-Cortol	14.8	9.7	13.2	8.7
(α + β)-Cortolone	12.3	7.5	11.8	7.1

^1^ CV-coefficient of variation.

**Table 2:** Comparison of concentrations (mg/24 h urine) of nineteen steroid hormones analyzed in samples from negative controls, patients with non-functioning adrenal incidentalomas, and patients with Cushing’s syndrome, values given as medians and interquartile ranges and the results of ANOVA Kruskal–Wallis test, followed by Dunn’s test (statistical significance level at *p* < 0.05).

Compound	Negative Control (*n* = 37)	Adrenal Incidentaloma (*N* = 25)	Cushing’s Syndrome (*n* = 16)	ANOVA Kruskal-Wallis Test Followed by Dunn’s Test
Androsterone	0.79	0.78	0.85	*H* = 15.21
	(0.76–0.83)	(0.7–0.81)	(0.83–0.91)	*p* < 0.001
Etiocholanolone	0.92	0.98	1.58	*H* = 38.66
	(0.88–0.97)	(0.89–0.99)	(1.5–1.74)	*p *< 0.0001
11-Keto-androsterone	0.16	0.16	0.18	*H* = 3.81
	(0.12–0.19)	(0.13–0.19)	(0.17–0.20)	*p* < 0.15
11-Hydroxy-androsterone	0.38	0.35	0.34	*H* = 4.75
	(0.34–0.44)	(0.29–0.44)	(0.31–0.38)	*p* < 0.09
11-Hydroxy-etiocholanolone	0.19	0.16	0.19	*H* = 4.05
	(0.16–0.22)	(0.15–0.19)	(0.19–0.21)	*p* < 0.13
Androstenetriol	0.09	0.09	0.09	*H* = 2.35
	(0.07–0.1)	(0.07–0.11)	(0.08–0.13)	*p* < 0.31
Pregnanetriol	1.34	1.3	1.43	*H* = 6.25
	(1.13–1.53)	(1.06–1.55)	(1.37–1.58)	*p* < 0.07
Tetrahydro-11-deoxycortisol	0.15	0.18	0.15	*H* = 3.36
	(0.13–0.19)	(0.16–0.22)	(0.14–0.17)	*p* < 0.19
11-Keto-pregnanetriol	0.34	0.31	0.41	*H* = 9.47
	(0.24–0.38)	(0.26–0.36)	(0.36–0.45)	*p* < 0.09
Pregnenetriol	0.22	0.24	0.26	*H* = 9.33
	(0.18–0.25)	(0.18–0.24)	(0.24–0.3)	*p* < 0.009
Tetrahydrocortisone	3.45	3.26	2.18	*H* = 34.92
	(3.12–3.57)	(3.0–3.56)	(2.11–2.35)	*p* < 0.0001
Tetrahydro-11-dehydrocorticosterone	0.31	0.27	0.27	*H* = 9.09
	(0.28–0.34)	(0.25–0.33)	(0.25–0.29)	*p* < 0.07
Tetrahydrocorticosterone	0.42	0.76	1.82	*H* =50.65
	(0.28–0.49)	(0.63–0.95)	(1.72–1.98)	*p* < 0.0001
allo-Tetrahydrocorticosterone	0.3	0.27	0.33	*H* =3.03
	(0.23–0.31)	(0.18–0.41)	(0.28–0.40)	*p* < 0.22
Tetrahydrocortisol	1.98	2.46	3.51	*H* = 51.34
	(1.78–2.05)	(2.31–2.82)	(3.20–3.71)	*p* < 0.0001
allo-Tetrahydrocortisol	0.85	1.08	1.86	*H* = 34.92
	(0.72–1.02)	(0.83–1.23)	(1.74–2.01)	*p* < 0.0001
α-Cortol	0.68	0.88	1.58	*H* = 50.49
	(0.63–0.74)	(0.75–1.04)	(1.45–1.78)	*P* < 0.0001
(α + β)-Cortolone	0.53	0.54	0.45	*H* = 5.69
	(0.44–0.67)	(0.45–0.61)	(0.39–0.47)	*P* < 0.06

**Table 3:** The results of Mann-Whitney’s *U* test performed for concentrations (mg/24 h urine) of nineteen steroid hormones analyzed in samples from patients with adrenal incidentaloma vs. control and Cushing’s syndrome patients vs. control (statistical significance level at *p* < 0.05).

Compound	Adrenal Incidentaloma vs. Control	Cushing’s Syndrome vs. Control
Androsterone	*U* = 401.50; *p* < 0.39	*U* = 111.00; *p* < 0.0001
Etiocholanolone	*U* = 378.50; *p* < 0.23	*U* = 0.00; *p* < 0.0001
11-Keto-androsterone	*U* = 458.50; *p* < 0.96	*U* = 215.00; *p* < 0.12
11-Hydroxy-androsterone	*U* = 380.00; *p *< 0.24	*U* = 177.00; *p* < 0.02
11-Hydroxy-etiocholanolone	*U* = 364.50; *p* < 0.16	*U* = 280.00; *p* < 0.76
Androstenetriol	*U* = 437.00; *p* < 0.72	*U* = 215.50; *p* < 0.12
Pregnanetriol	*U* = 454.00; *p* < 0.91	*U* = 180.00; *p* < 0.03
Tetrahydro-11-deoxycortisol	*U* = 366.00; *p* < 0.17	*U* = 281.50; *p* < 0.79
11-Keto-pregnanetriol	*U* = 436.50; *p* < 0.71	*U* = 161.50; *p* < 0.01
Pregnenetriol	*U* = 349.50; *p *< 0.11	*U* = 132.50; *p* < 0.0001
Tetrahydrocortisone	*U* = 411.00; *p* < 0.46	*U* = 7.00; *p* < 0.0001
Tetrahydro-11-dehydrocorticosterone	*U* = 341.00; *p* < 0.08	*U* = 140.0; *p* < 0.0001
Tetrahydrocorticosterone	*U* = 147.50; *p* < 0.0001	*U* = 0.00; *p* < 0.0001
allo-Tetrahydrocorticosterone	*U* = 438.00; *p* < 0.73	*U* = 194.50; *p* < 0.05
Tetrahydrocortisol	*U* = 131.00; *p* < 0.0001	*U* = 0.00; *p* < 0.0001
allo-Tetrahydrocortisol	*U* = 308.00; *p* < 0.003	*U* = 19.50; *p* < 0.0001
α-Cortol	*U* = 140.00; *p* < 0.0001	*U* = 0.00; *p* < 0.0001
(α + β)-Cortolone	*U* = 445.50; *p* < 0.81	*U* = 192.00; *p* < 0.04

**Table 4:** Results of multiple linear regression analysis of potential confounders (statistical significance level at *p* < 0.05; *R* = 0.79).

Variable	β ^1^	Standard Error of β ^1^	B ^2^	Standard Error of B ^2^	*T*	*p*-Value
Intercept			2.16	1.61	1.34	0.19
Androsterone	−0.03	0.11	−0.17	0.60	−0.28	0.78
Etiocholanolone	−0.98	0.20	−2.87	0.60	−4.79	0.000
11-Keto-androsterone	−0.21	0.10	−3.73	1.67	−2.23	0.03
11-Hydroxy-androsterone	−0.25	0.12	−2.59	1.23	−2.11	0.04
11-Hydroxy-etiocholanolone	0.14	0.11	2.40	1.87	1.29	0.20
Androstenetriol	0.20	0.10	2.48	1.30	1.90	0.06
Pregnanetriol	0.16	0.09	0.28	0.16	1.71	0.09
Tetrahydro-11-deoxycortisol	0.13	0.09	2.15	1.53	1.41	0.17
11-Keto-pregnanetriol	−0.17	0.09	−0.93	0.51	−1.83	0.07
Pregnenetriol	0.22	0.09	1.54	0.63	2.44	0.02
Tetrahydrocortisone	−0.02	0.14	−0.02	0.21	−0.11	0.91
Tetrahydro-11-dehydrocorticosterone	−0.25	0.09	−3.81	1.38	−2.77	0.01
Tetrahydrocorticosterone	0.13	0.22	0.20	0.33	0.60	0.55
allo-Tetrahydrocorticosterone	0.16	0.09	1.17	0.71	1.64	0.11
Tetrahydrocortisol	0.54	0.17	0.68	0.21	3.18	0.000
allo-Tetrahydrocortisol	−0.04	0.14	−0.07	0.23	−0.32	0.75
α-Cortol	0.57	0.19	1.26	0.43	2.91	0.01
(α + β)-Cortolone	−0.11	0.10	−0.33	0.28	−1.17	0.25
Sex	0.05	0.10	0.09	0.18	0.52	0.61

^1^ standardized regression coefficient; ^2^ unstandardized (raw) regression coefficient.

**Table 5:** Classification power of the linear discriminant analysis (LDA) method.

Classes	Classification (%)	Predicted Groups		
		Negative Control	Adrenal Incidentaloma	Cushing’s Syndrome
Negative control	91.89	34	3	0
Adrenal incidentaloma	80	5	20	0
Cushing’s syndrome	100	0	0	16
Total	89.74	39	23	16

**Table 6:** Predictive power of the LDA method after applying leave-one-out (LOO) cross-validation.

Classes	Classification (%)	Predicted Groups (Cross-Validation)		
		Negative Control	Adrenal Incidentaloma	Cushing’s Syndrome
Negative control	83.78	31	6	0
Adrenal incidentaloma	76	6	19	0
Cushing’s syndrome	100	0	0	16
Total	86.59	37	25	16

## Supplementary Materials

Supplementary materials can be found at www.mdpi.com/1422-0067/18/2/294/s1.
