# Supplementary material for: Metabolomic Biomarkers in Urine of Cushing’s Syndrome Patients
Source: Int J Mol Sci. 2017 Jan 29;18(2):294. doi: 10.3390/ijms18020294 (PMC5343830; doi:10.3390/ijms18020294)
Supplement: Supplementary file 1 [file ijms-18-00294-s001.pdf]

Supplementary Materials: Metabolomic Biomarkers in Urine of Cushing's Syndrome Patients

Alicja Kotłowska, Tomasz Puzyn, Krzysztof Sworczak, Piotr Stepnowski and Piotr Szefer

Table S1. Metadata obtained for patients. All the results apart from gender are expressed as medians (5th, 95th percentile).

Metadata	Adrenal Incidentaloma (*n* = 25)	Cushing's Syndrome (*n* = 16)
Age (years)	49 (43, 57)	51 (44, 58)
Gender (%)	72% females<br>28% males	68.75% females<br>31.25% males
Height (cm)	163 (157, 176)	167.5 (158, 178)
Weight (kg)	77 (71, 89)	86 (74.5, 101)
BMI (kg/m^2)	28.9 (27.9, 30.3)	30.8 (29.9, 33)
UFC (nmol/24 h) [1]	112 (86, 144)	905 (690, 1409)
Serum cortisol after DXM suppression test (nmol/L) [2]	66 (36, 85)	279 (170, 367)

[1] UFC normal range: 12–330 nmol/24 h, values > 330 nmol/24 h indicated hypercortisolism; [2] Serum cortisol after dexamethasone (DXM) suppression test: concentrations < 50 nmol/L excluded hypercortisolism, intermediate cut-off point of 94 nmol/L indicated subclinical hypercortisolism, >140 nmol/L indicated hypercortisolism.
